# Emerging Terbinafine Resistant Trichophyton Dermatophytosis, Testing Options and Alternative Treatments: A Systematic Review

**DOI:** 10.1111/ajd.14575

**Published:** 2025-07-24

**Authors:** Thuvarahan Jegathees, Zachary P. Holmes, Catherine Martin, Cindy Kalai, Catherine Voutier, Denis Spelman, Gemma Robertson, Johannes S. Kern

**Affiliations:** ^1^ Department of Dermatology Alfred Health Melbourne Victoria Australia; ^2^ Westmead Clinical School The University of Sydney Sydney New South Wales Australia; ^3^ School of Translational Medicine Monash University Melbourne Victoria Australia; ^4^ School of Public Health and Preventive Medicine Monash University Melbourne Victoria Australia; ^5^ Royal Melbourne Hospital Library Parkville Victoria Australia; ^6^ Department of Infectious Diseases, Microbiology Alfred Health Melbourne Victoria Australia; ^7^ Melbourne Pathology Collingwood Victoria Australia

## Abstract

Dermatophytosis is a common superficial fungal infection of the skin, most often caused by dermatophytes from the *Trichophyton* genus. Terbinafine, which inhibits squalene epoxidase (SQLE), is widely used as first line treatment. However, resistance to terbinafine is increasing globally, including recent reports in Australia, with origins suspected to trace back to South Asia. Antifungal susceptibility testing is not routinely available in Australia, and globally, there are no standardised breakpoints for traditional culture‐based methods. Although SQLE gene mutations have been associated with terbinafine resistance, a major research gap exists in the clinical interpretation of these mutations due to a lack of correlation between MIC values, genetic mutations and clinical treatment outcomes. This gap hampers the ability to guide clinical decision‐making. Therefore, our objective was to assess the global prevalence of terbinafine‐resistant *Trichophyton* infections, identify the most clinically relevant resistance testing methods, and determine effective alternative treatment options. The study was designed by systematic review. Ovid MEDLINE, Ovid Embase, Cochrane Database of Systematic Reviews (CDSR), Cochrane's Trials database (CENTRAL), Global Health (CABI), Trials registries, along with Google Scholar and Web of Science (WoS) for the tracking of included articles. Published human studies in English from 2000 to 2023 of Terbinafine‐resistant Trichophyton Dermatophytosis with confirmed antifungal susceptibility tests and genotyping of dermatophytes, as well as details of effective alternative treatments. Identified cases were independently screened by two authors on the basis of predetermined criteria. Thirty four studies reported 743 samples for which mutation data of the SQLE gene and minimum inhibitory concentration (MIC) were available. Twenty three studies reported on 149 patients who had used terbinafine with available MIC data. Of these, 94 cases demonstrated evidence of clinical resistance to terbinafine with confirmed SQLE genotyping and MIC data. Seven studies reported on 13 cases of clinical resistance to terbinafine with a reported MIC and a successful alternative therapy. There are no published MIC breakpoints for terbinafine resistance in antifungal resistance testing, creating significant challenges for clinical interpretation. This study suggests that an estimate of a provisional MIC threshold for resistance is calculated to be 1.69 μg/mL. Importantly, SQLE mutation data, particularly the presence of F397L, L393F and A448T shows a robust association with clinical resistance to terbinafine (odds ratio: 7.58; 14.0, 7.78, respectively). Routine SQLE mutation testing in cases of suspected terbinafine‐resistant dermatophytosis could enhance diagnostic accuracy and inform more effective, timely treatment decisions. Identifying specific mutations may guide clinicians in selecting alternative antifungal agents earlier in the treatment course, reducing morbidity and improving outcomes. Systematic review was registered with PROSPERO.

**Trial Registration:** PROSPERO number: CRD42022382880

## 1 Introduction

Dermatophytosis is a prevalent superficial fungal infection affecting the skin and its keratinised structures, with an estimated global prevalence of 20%–25%, representing approximately 1.65 billion cases, according to the Global Burden of Disease (GBD) 2019 study [1]. Dermatophytes from the genus Trichophyton, including *T. rubrum*, *T. interdigitale* and *T. mentagrophytes*, represent a primary etiological factor of superficial mycoses [2]. Clinically, the condition presents as annular, erythematous, scaly skin plaques, and may involve nail discoloration, thickening and eventual destruction [3]. Anthropophilic dermatophytes exhibit a high degree of adaptation to human skin, often inducing a less severe inflammatory response compared to zoophilic variants [4]. Although rarely life‐threatening, cutaneous infections can negatively affect quality of life through pruritus and cosmetic concerns, and in some cases, invasive disease among immunocompromised individuals [5]. Terbinafine, a squalene epoxidase (SQLE) enzyme inhibitor, is commonly prescribed as a first‐line treatment for trichophyton dermatophytosis due to its proven efficacy. This enzyme is indispensable for the biosynthesis of ergosterol, a critical fungal cell membrane component [2, 6]. Since 2003, a troubling increase in reports of terbinafine‐resistant Trichophyton infections has emerged worldwide [7, 8]. This trend has substantial clinical and economic implications. Resistant infections are often refractory to first‐line therapy, leading to prolonged disease duration, increased healthcare costs, repeated medical consultations, and the use of broader‐spectrum or combination antifungal therapies, which may have higher toxicity profiles or reduced efficacy.

Epidemiologically, terbinafine‐resistant strains have been reported in various countries including India, Japan, Iran, Germany, Switzerland, Poland, Belgium and Denmark [6], with more recent cases surfacing in Australia [9]. Notably, resistant infections have also been documented in patients without a travel history, underscoring the potential for local transmission. This raises public health concerns, especially in countries like Australia, where individuals of South Asian descent, a region hypothesised to be the origin of resistance [10], constitute a significant portion of the overseas‐born population (approximately 15%) [11].

In cases of refractory dermatophytosis, terbinafine resistance is frequently associated with specific point mutations in the SQLE gene, notably at positions L393F and F397L [12]; however, the interpretation of mutations in clinical practice has been hindered by a lack of clinical resistance data [10, 11]. Additionally, antifungal susceptibility testing is not routinely conducted within Australia, partly due to the slow proliferation rate of dermatophytes, high contamination risk in cultures, and the absence of established clinical breakpoints necessary for interpreting MIC results [9, 13]. Additionally, the variability of methods used for antifungal susceptibility testing presents challenges; methodologies such as agar disc diffusion, macro‐broth, and micro‐broth dilution tests differ in terms of inoculum density, incubation conditions, and endpoint criteria for fungal growth [13].

Critically, the current literature lacks integrated clinical data correlating SQLE mutation profiles, MIC values, and actual treatment outcomes. This represents a significant gap that hinders the development of standardised diagnostic and therapeutic guidelines for terbinafine‐resistant dermatophytosis.

This systematic review evaluates the global prevalence and distribution of terbinafine‐resistant *Trichophyton* infections, examines current resistance testing methods—including MIC‐based and genetic approaches—and identifies effective alternative treatments. A systematic review is well suited to this topic as it synthesises diverse data sources, clarifies inconsistencies and highlights clinically relevant patterns. By integrating molecular, microbiological and clinical findings, this review addresses key evidence gaps and provides practical insights to inform diagnosis, treatment and future research.

## 2 Methods

The systematic review was executed adhering to the guidelines established by the Preferred Reporting Items for Systematic Reviews and Meta‐Analyses (PRISMA) [13]. The protocol for this systematic review was duly registered with PROSPERO (International Prospective Register of Systematic Reviews); registration number: CRD42022382880.

### 2.1 Search Strategy

To identify relevant studies, we searched the following databases: MEDLINE(R) ALL, Embase and Global Health (all on the Ovid platform) and the Cochrane Central Register of Controlled Trials (CCTR) via the Cochrane Library hosted by Wiley. The search strategy was built using text and index terms or gold standard articles and run in all databases up to January 2023. No database filters were used. The clinical trials registries produced by the United States Government and the World Health Organisation were also searched (clinicaltrials.gov) and the International Clinical Trials Registry Platform. EndNote x9, produced by Clarivate, was used to download all database results and retrieve full text. The EndNote xml file was uploaded into Covidence, which auto‐removed duplicate records. Records that were duplicates but not marked by Covidence as such were indicated manually.

A full description of the search strategy is listed in Appendix S1. Search terms included drug terms for terbinafine, drug resistance and specific types of trichophyton infection. A mix of thesaurus terms and keywords was used.

### 2.2 Review of Studies

Two evaluators (T.J and Z.P.H) conducted an independent assessment of the retrieved studies utilising Covidence, facilitating blinded screening of titles, abstracts and full‐text articles. Discrepancies were resolved through consensus.

### 2.3 Eligibility Criteria

The selected studies adhered to the following inclusion criteria: (i) observational studies involving human subjects, encompassing retrospective cohort studies, case series and case reports; (ii) articles published from the year 2000 onward; (iii) full‐text articles that have undergone peer review; (iv) studies providing data on antifungal susceptibility testing. The exclusion criteria were as follows: (i) studies focused exclusively on non‐human cases of dermatophytosis; (ii) research involving only non‐trichophyton cases of dermatophytosis; (iii) studies that provide antifungal susceptibility data solely for trichophyton cases of dermatophytosis but do not include either clinical usage data for terbinafine or SQLE mutation data; (iv) articles not authored in English; (v) studies not addressing antifungal susceptibility to terbinafine; (vi) reviews, editorials, or letters.

### 2.4 Data Extraction

Data extracted from the selected studies encompassed the following: (i) characteristics of the study including the first author, year of publication and sample size; (ii) demographic and clinical characterisation such as age, sex, country of residence, Trichophyton subtype, clinical application and dosage of terbinafine, as well as clinical application and dosage of alternative therapies and presence of SQLE mutation if applicable; (iii) outcomes including antifungal susceptibility testing employed, results of terbinafine antifungal susceptibility testing and clinical resistance to terbinafine. Data extraction was performed by one reviewer and its validity was ascertained by a second reviewer who cross‐checked data entered from all included papers.

### 2.5 Statistical Analysis

The analysis was conducted at the individual level. The majority of the studies comprised right and/or left censored MIC data. Therefore, interval regression was employed, using Stata's intreg command. This method is appropriate for interval‐censored data, where MIC values may fall below a lower detection limit or exceed an upper detection threshold, resulting in partially observed intervals rather than exact values. The data was set up as interval data, with the lower endpoint data in one variable and the upper endpoint data in a second. In Stata, for left‐censored data the lower endpoint is represented by a missing value, and for the right‐censored data, the upper endpoint is represented by a missing value. The standard errors reported take into account the clustering of each study. Due to the substantial skewness of MIC values, a logarithmic transformation was applied for analysis, followed by back transformation to yield a geometric mean, which are interpreted as for ordinary least‐squares regression. The estimated MIC values were compared using the wild type (WT) as the reference value. Mutations with small sample sizes were not included in comparisons. P values less than 0.05 were regarded as statistically significant. All statistical tests were performed as two‐sided. Stata version 17 (StataCorp. 2021. *Stata Statistical Software: Release 18*. College Station, TX: StataCorp LLC) was used for the analysis.

## 3 Results

**FIGURE 1:**
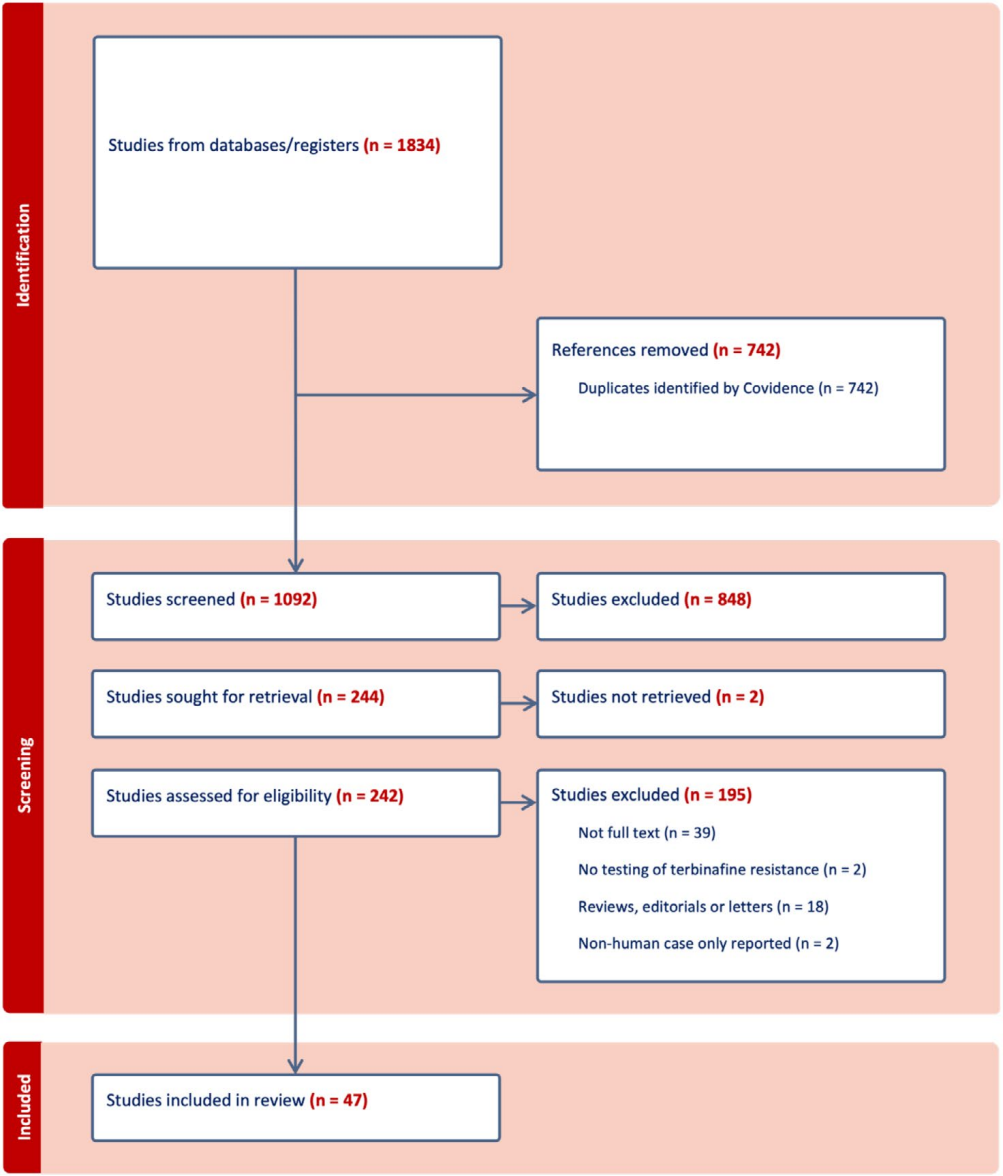
PRISMA diagram of included studies.

A total of 1834 records were identified through database searches, from which 742 duplicates were excluded. Consequently, 1092 articles underwent initial screening, with 242 articles subjected to full‐text assessment for eligibility, resulting in 47 articles meeting the inclusion criteria (Figure 1). The final list of included articles is provided in Appendix S2. Among these, 34 studies reported a total of 743 samples for which mutation data of the SQLE gene and MIC data were available (Table 1). Furthermore, 23 studies detailed 149 patients who had either utilised topical, oral terbinafine, or a combination thereof, with corresponding MIC data available (Table 3). From this subset, 94 cases presented evidence of clinical resistance to either topical or oral terbinafine, corroborated by confirmed SQLE genotyping and MIC data (Table 4). Additionally, 7 studies reported on 13 cases of clinical resistance to either topical or oral terbinafine, with documented MIC and subsequent successful alternative therapy (Table 5).

**TABLE 1:** Study characteristics of papers reporting on both MIC values and SQLE mutations.

Author	Country	Type of study	Number of participants	MIC method
Abastabar et al. [14]	Iran	Prospective	2100	CLSI
Astvad et al. [15]	Denmark	Case series	63	EUCAST
Bidaud et al. [16]	India	Case series	16	EUCAST
Brasch et al. [17]	Germany	Case series	3	EUCAST
Dellière et al. [18]	France	Case series	9	EUCAST
Durdu et al. [19]	Türkiye	Case series	2	EUCAST
Ebert et al. [3]	India	Case series	297	CLSI
Gaurav et al. [20]	India	Case series	6	CLSI
Hiruma et al. [21]	Japan	Case series	5	CLSI
Hsieh et al. [22]	Switzerland	Case series	2	CLSI
Jabet et al. [23]	France	Case series	10	EUCAST
Kakurai et al. [24]	Japan	Case report	1	CLSI
Kano et al. [25]	Japan	Case series	2	CLSI
Kano et al. [26]	Japan	Case report	1	CLSI
Khurana et al. [27]	India	Prospective	30	CLSI
Kimura et al. [28]	Japan	Case report	1	CLSI
Klinger et al. [29]	Switzerland	Case series	2	Other
Moreno‐Sabater et al. [30]	France	Case series	23	EUCAST
Nenoff et al. [31]	Germany	Case series	29	CLSI
Ngo et al. [32]	Vietnam	Case report	1	EUCAST
Noguchi et al. [33]	Japan	Case series	30	CLSI
Noguchi, Matsumoto, Kubo, Kimura, Hiruma, Yaguchi et al. [34]	Japan	Case report	1	CLSI
Noguchi, Matsumoto, Kubo, Kimura, Hiruma, Tanaka et al. [35]	Japan	Case report	1	CLSI
Rudramurthy et al. [36]	India	Case series	20	CLSI
Sacheli et al. [37]	Belgium	Prospective survey	5	EUCAST
Sardana et al. [38]	India	Prospective cross‐sectional analysis	21	CLSI
Saunte et al. [10]	Denmark	Case series	14	EUCAST
Shankarnarayan et al. [2]	India	Prospective	15	CLSI
Singh et al. [39]	India	Case series	30	CLSI
Singh et al. [40]	India	Case series	61	CLSI
Siopi et al. [41]	Greece	Case series	5	EUCAST
Taghipour et al. [42]	Iran	Case series	10	CLSI
Kong et al. [43]	India/China	Case series	62	EUCAST
Łagowski et al. [44]	Poland	Case series	7	CLSI

### 3.1 SQLE Mutation and Reported MIC

A total of 34 studies encompassing 743 samples have documented both the SQLE mutation and the MIC of Terbinafine across several countries, specifically India (*n* = 10), France (*n* = 3), Japan (*n* = 8), Iran (*n* = 2), Denmark (*n* = 2), Germany (*n* = 2), Switzerland (*n* = 2), Türkiye (*n* = 1), Vietnam (*n* = 1), Belgium (*n* = 1), Poland (*n* = 1) and China (*n* = 1) (refer to Table 1). The research comprised a diverse array of methodological approaches, including prospective studies (*n* = 5), retrospective case series/cohort studies (*n* = 22) and case reports (*n* = 7). Predominantly, these studies reported the MIC based on the guidelines of the Clinical & Laboratory Standards Institute (CLSI) (21/34) or the European Committee on Antimicrobial Susceptibility Testing (EUCAST) (12/34).

The most frequently identified SQLE genotype in these investigations was F397L (51.7%), WT (18.8%), A448T (12.7%), L393F (7.9%) and L393S (3.5%) mutations (Table 2). MIC estimates were performed for the five most prevalent SLQE mutations alongside the wild‐type genotype using interval regression analysis. This methodological approach was necessitated due to some studies reporting MIC values as left‐censored, others as right‐censored, while some studies did not report MIC values at all. The MIC values for L393F (46.0 μg/mL), F397L (11.9 μg/mL) and L393S (0.9 μg/mL) were substantially higher compared to that of the wild‐type genotype (0.05 μg/mL).

**TABLE 2:** Estimated MIC value of SQLE mutations. Data collected from 34 studies with 743 samples reported on both SQLE mutation and the MIC of Terbinafine. MIC values determined through interval regression analysis.

Factor	*n* (%)	MIC estimate (95% CI)	*p*‐value compared to WT
*N*	743		
Mutation			
WT	140 (18.8%)	0.05 (0.01, 0.22)	
F397L	384 (51.7%)	11.9 (5.5, 26.1)	< 0.001
A448T	94 (12.7%)	0.1 (0.0, 0.2)	0.89
L393F	59 (7.9%)	46.0 (11.7, 181.2)	< 0.001
L393S	26 (3.5%)	0.9 (0.5, 1.7)	< 0.001
K276N	14 (1.9%)	0.0 (0.0, 0.0)	0.50
H440Y	5 (0.7%)		
L419F	3 (0.4%)		
S443P	3 (0.4%)		
A1223T	2 (0.3%)		
C1255T	2 (0.3%)		
F397I	2 (0.3%)		
F397	1 (0.1%)		
F397L/A448T	1 (0.1%)		
F398	1 (0.1%)		
F415S	1 (0.1%)		
F415V	1 (0.1%)		
I121M	1 (0.1%)		
I479V	1 (0.1%)		
L437P	1 (0.1%)		
T414H	1 (0.1%)		

### 3.2 Clinical Resistance and SQLE Mutation

A total of 23 studies (Table 3) documented patients who had been treated with either topical, oral terbinafine, or both, incorporating MIC data (*n* = 149) or MIC data in conjunction with SQLE genotyping (*n* = 102) (Table 4). Among these, 121 individuals (82%) exhibited clinical resistance, and within this group, 94 cases included SQLE genotyping alongside MIC data. Data were gathered from studies conducted in India (3 studies, 37 patients), Japan (5 studies, 15 patients), Denmark (3 studies, 16 patients), Germany (2 studies, 31 patients), France (2 studies, 14 patients), Canada (1 study, 13 patients), Türkiye (1 study, 2 patients), Iran (1 study, 2 patients), Poland (1 study, 9 patients), Switzerland (1 study, 1 patient), China (1 study, 1 patient), Austria (1 study, 1 patient) and the USA (1 study, 1 patient). An early study noted increased MIC levels in Trichophyton as early as 2003 [2].

**TABLE 3:** Study characteristics of papers reporting patients who had been exposed to either topical, or oral terbinafine, or a combination of the two with MIC data.

Study	Country	Resistant/*N*	Minimum MIC	Maximum MIC
Brasch et al. [17]	Germany	1/3	< 0.06	< 0.06
Dellière et al. [18]	France	6/8	0.03	4
Durdu et al. [19]	Türkiye	2/2	> 16	> 16
Firooz et al. [45]	Iran	2/2	> 4	> 4
Gaurav et al. [20]	India	6/6	0.125	8
Gnat et al. [46]	Poland	9/9	0.5	2
Gu et al. [47]	USA	1/1	> 0.5	> 0.5
Gupta et al. [48]	Canada	18/18	0.007	0.03
Hiruma et al. [49]	Japan	3/3	32	> 32
Hsieh et al. [22]	Switzerland	1/1	> 1	> 1
Jabet et al. [23]	France	3/6	0.06	> 8
Kakurai et al. [24]	Japan	1/1	32	32
Kano et al. [26]	Japan	1/1	32	32
Khurana et al. [27]	India	9/30	0.5	> 32
Khurana et al. [50]	India	1/1	32	32
Kimura et al. [28]	Japan	1/1	> 32	> 32
Mukherjee et al. [8]	Austria	1/1	4	4
Nenoff et al. [31]	Germany	29/29	< 0.2	16
Noguchi et al. [33]	Japan	9/9	> 32	> 32
Saunte et al. [10]	Denmark	14/14	0.125	> 8
Schosler et al. [51]	Denmark	1/1	4	4
Liu et al. [52]	China	1/1	< 0.5	< 0.5
Digby et al. [53]	Denmark	1/1	> 4	> 4

**TABLE 4:** Clinical characteristics of 148 patients from 23 studies which had reported exposure to either topical or oral terbinafine, or a combination of the two with MIC data. SQLE mutation data was available for 102 patients.

Factor	No	Yes
*N*	27	121
Year		
2003	0 (0.0%)	19 (15.7%)
2016	0 (0.0%)	1 (0.8%)
2018	21 (77.8%)	10 (8.3%)
2019	0 (0.0%)	15 (12.4%)
2020	0 (0.0%)	45 (37.2%)
2021	1 (3.7%)	18 (14.9%)
2022	5 (18.5%)	11 (9.1%)
2023	0 (0.0%)	2 (1.7%)
Country		
Austria	0 (0.0%)	1 (0.8%)
Canada	0 (0.0%)	18 (14.9%)
China	0 (0.0%)	1 (0.8%)
Denmark	0 (0.0%)	16 (13.2%)
France	5 (18.5%)	9 (7.4%)
Germany	1 (3.7%)	30 (24.8%)
India	21 (77.8%)	16 (13.2%)
Iran	0 (0.0%)	2 (1.7%)
Japan	0 (0.0%)	15 (12.4%)
Poland	0 (0.0%)	9 (7.4%)
Switzerland	0 (0.0%)	1 (0.8%)
Türkiye	0 (0.0%)	2 (1.7%)
United States	0 (0.0%)	1 (0.8%)
SQLE mutation		
WT	7 (44%)	9 (11%)
A448T	1 (6%)	10 (12%)
F397L	4 (25%)	39 (46%)
L393F	1 (6%)	18 (21%)
F415S	0 (0%)	1 (1%)
H440Y	0 (0%)	1 (1%)
I121M	0 (0%)	1 (1%)
K276N	2 (12%)	0 (0%)
L393S	1 (6%)	4 (5%)
Q408L	0 (0%)	1 (1%)
V444T	0 (0%)	1 (1%)
Species		
*T. indotineae*	3 (11.1%)	6 (5.0%)
*T. interdigitale*	21 (77.8%)	13 (10.7%)
*T. mentagrophytes*	3 (11.1%)	51 (41.0%)
*T. rubrum*	0 (0.0%)	48 (39.7%)
*T. tonsurans*	0 (0.0%)	2 (1.7%)
Previous use of terbinafine		
Both	0 (0.0%)	38 (31.4%)
Oral	25 (100.0%)	78 (64.5%)
Topical	0 (0.0%)	5 (4.1%)
Laboratory method		
CLSI	0 (0.0%)	64 (57.7%)
EUCAST	4 (66.7%)	22 (19.8%)
Other	2 (33.3%)	25 (22.5%)

The studies implemented various methodologies for reporting the MIC values such as those based on the CLSI, EUCAST and alternative micro and macro broth dilution techniques. The lowest MIC value associated with clinical resistance was reported at 0.03 μg/mL, whereas the highest was indicated as greater than 32 μg/mL (Table 3).

Interval regression analysis was employed to determine a potential MIC breakpoint for clinical resistance, given that the data were subject to left‐ and right‐censored limitations. The data needed to be log‐transformed due to non‐normal distribution for analysis purposes and subsequently back‐transformed, yielding a geometric mean. Out of the 23 studies, 19 reported a 100% rate of clinical resistance. Of 128 individuals with an established MIC value, 121 (82%) exhibited clinical resistance. The estimated MIC threshold for resistance is calculated to be 1.69 μg/mL (95% confidence interval [CI]: 0.89, 3.23, *p* = 0.11). When taking study clustering into account, the estimated MIC value for resistance remains 1.69 μg/mL (95% CI: 0.22, 13.26, *p* = 0.62).

The predominant SQLE genotypes identified in these studies were mutations F397L (29.1%), L393F (12.8%), wild‐type (WT) (10.8%), A448T (7.4%) and L393S (3.4%) (Table 4). Mutations F397L (Odds Ratio [OR] 7.58, CI: 1.82, 31.57, *p* = 0.005), L393F (OR 14.0, CI: 1.49, 131.89, *p* = 0.02) and A448T (OR 7.78, CI: 0.80, 76.09, *p* = 0.08) were more frequently associated with clinical resistance when compared to the wild‐type phenotype. In contrast, the L393S mutation did not indicate a clinical resistance to terbinafine (OR 3.11, CI: 0.28, 34.42, *p* = 0.35).

### 3.3 Alternative Therapies

Seven studies documented 13 instances of clinical resistance to either topical or oral terbinafine, including a reported MIC and an effective alternative therapeutic approach (Table 5). The MIC range observed across these instances was 0.125–32 μg/mL. Genotypic data concerning the SQLE gene was available for only two cases, both of which exhibited the F397L mutation. Successful treatment outcomes were achieved through itraconazole (*n* = 6), griseofulvin (*n* = 4), lanoconazole (*n* = 1) and fosravuconazole (*n* = 1).

**TABLE 5:** Clinical Characteristics of 13 patients reported from 7 studies which reported clinical resistance to either topical or oral terbinafine with a reported MIC where a successful alternative therapy was used.

Study	Country	Number of patients	Age ± SD (years)	Terbinafine exposure	MIC (μg/mL)	*SQLE* PCR	Alternative therapies
Digby et al. [53]	Denmark	1	62	PO 250 mg/day for 2 months	> 4^E^	Not reported	Itraconazole
Schosler et al. [51]	Denmark	1	9	Topical and oral	4^E^	Not reported	Itraconazole (100 mg/d) for 3 week
Gu et al. [47]	United States	1	45	Yes	> 0.5^C^	Not reported	Itraconazole
Gaurav et al. [20]	India	6	38.5 ± 14.1	Yes	0.125‐8^C^	Not reported	Griseofulvin ×4, itraconazole ×2
Noguchi et al. [34]	Japan	1	81	Topical for 2 years	32^C^	F397L	Fosravuconazole
Kano et al. [26]	Japan	1	81	Topical	32^C^	Not reported	Lanoconazole
Durdu et al. [19]	Türkiye	2	26 ± 1	PO 250 mg/day for 2 months	> 16^M^	F397L	Itraconazole

Abbreviations: C = CLSI, E = EUCAST, M = microbroth.

## 4 Discussion

There is an alarming rise in not only the number of cases of trichophyton dermatophytosis, but also a rise in the number of recurrent and clinically resistant cases [2]. Large outbreaks were first described across the Indian subcontinent; however, there are now reported cases in patients without a travel history, which highlights local human‐to‐human transmission [3, 54]. This reflects a concerning global epidemiological shift and a need for more robust surveillance. In Australia, terbinafine‐resistant dermatophytosis has now been documented [9], with some reports that up to 18% of *trichophyton* dermatophytosis is resistant to terbinafine [55].

Several factors may be contributing to this rise, including poor prescribing practices. In Australia, terbinafine is a first line therapy for all forms of dermatophytosis. Topical formulations are available over the counter, and oral terbinafine is both affordable and subsidised under the Pharmaceutical Benefits Scheme (PBS) for severe onychomycosis [9]. These factors may be inadvertently promoting overuse and under‐supervised treatment, increasing the risk of resistance development. This study highlights the paucity of studies reporting on clinical antifungal resistance confirmed by in vitro MIC data or SQLE mutation data. Furthermore, there is a lack of studies reporting on successful alternatives therapies for treatment failure with terbinafine that are supported by corresponding susceptibility data.

Although the CLSI and EUCAST offer methods for determining antifungal susceptibility, neither of these methods specifies a breakpoint for terbinafine sensitivity, and neither method is routinely available in Australia [9]. One key finding of this review is the estimated MIC threshold for clinical resistance to terbinafine. Using interval regression analysis across reported methodologies, we propose that an MIC of > 1.69 μg/mL (95% CI: 0.22, 13.26, *p* = 0.62) may serve as an indicator of clinical resistance to terbinafine. However, this value lacks robustness with many studies reporting clinical resistance of trichophyton dermatophytosis to terbinafine with MIC values reported lower than this (Table 3). For example, Blanchard and colleagues [12] suggest a value of 0.015 μg/mL to indicate clinical resistance based upon the lowest MIC value reported in their experimental samples.

The variation in proposed thresholds may stem from differences in antifungal susceptibility testing protocols, inoculum concentration, incubation time and temperature, as well as the end‐point criteria for fungal growth. Additionally, MIC testing is limited by its in vitro nature, failing to account for patient‐specific factors such as drug absorption, distribution, comorbidities and adherence. As such, MIC values alone may be insufficient for guiding clinical decisions in dermatophytosis [56].

Given these limitations, this review also explored SQLE gene mutations as potential markers of resistance. The SQLE enzyme is required for the synthesis of ergosterol, a key component of the fungal cell membrane, which is inhibited by terbinafine. Mutations at L393F, F397L and L393S were associated with significantly elevated MICs (46.0, 11.9 and 0.9 μg/mL, respectively) compared to the wild‐type genotype (0.05 μg/mL) and clinical failure of terbinafine therapy, making them strong candidates for diagnostic markers (Table 2). Interestingly, although A448T demonstrated a much lower MIC (0.1 μg/mL; CI: 0.0–0.2), it was still linked with clinical resistance (Tables 2 and 4). This dissociation suggests that the presence of certain mutations may affect drug binding or ergosterol synthesis efficiency in ways not fully captured by MIC values alone. It also raises the possibility that it may be the species of Trichophyton or the mutations in the SQLE gene that may be more predictive of clinical resistance than MIC values alone. These findings provide new insight into the complexity of terbinafine resistance and highlight the need to incorporate molecular data alongside conventional MIC testing. Despite the promise of SQLE mutation testing as a diagnostic tool, several barriers impede its clinical implementation. These include the cost and technical complexity of molecular assays, lack of standardised protocols, and limited access to specialised laboratories, particularly in regional or resource‐limited settings. Notably, SQLE mutation testing is not available in Australia, highlighting a significant diagnostic gap and potential risk of under detection and treatment of resistant trichophytes.

The emergence of terbinafine resistance has prompted a re‐evaluation of treatment strategies. Among the alternative therapies reviewed, itraconazole and griseofulvin were most frequently reported as effective, particularly in cases involving T. indotineae, a strain now considered endemic in South Asia. Itraconazole's broad‐spectrum fungistatic activity, its mechanism of inhibiting lanosterol 14α‐demethylase, and higher tissue affinity in skin and nails may contribute to its success where terbinafine fails [54]. The Indian Association of Dermatology, Venereology and Leprosy (IADVL) now recommends higher doses and extended durations of itraconazole for recalcitrant cases [54, 57]. These findings reinforce the importance of adapting antifungal regimens based on emerging resistance patterns.

### 4.1 Limitations

With a paucity of literature in this globally important disease, our estimates of the MIC values for resistance and for each of the SQLE mutations may be limited. Data used in this study was from the published literature with variation in methodologies. In addition, there appears to be a large reporting bias, with reported clinical resistance in studies captured in this review (82%) being much higher than laboratory‐reported resistance (18%) taken as MIC > 0.5 μg/mL in a 2023 study from North America [55]. This may have impacted the odds ratios for resistance conferred by the detection of SQLE mutations.

## 5 Conclusions

While antimicrobial stewardship is widely acknowledged, antifungal stewardship remains insufficient, as evidenced by incidence of terbinafine‐resistant Trichophyton cases increasing globally, including in Australia. To enhance appropriate antifungal application, it is imperative to establish precise diagnostic guidelines and optimal treatment strategies, encompassing dosing, therapy duration and susceptibility testing. Prioritising epidemiological surveillance and educational initiatives is crucial to mitigate diagnostic delays and enhance adherence. Currently, there is an absence of standardised testing methods or MIC breakpoints for terbinafine resistance and few studies correlate in vitro susceptibility data with clinical outcomes. Moreover, the majority of published data originates from case reports or small observational studies, limiting generalisability. This study suggests that estimated MIC threshold for resistance is calculated to be 1.69 μg/mL, however, standardised testing methods would need to be established. The identification of SQLE mutations that correlate more consistently with treatment failure than MIC values offers a new avenue for refining diagnostic thresholds and resistance criteria. Mutations F397L, L393F and A448T (Odds Ratio: 7.58; 14.0, 7.78) more frequently associated with clinical resistance when compared to the wild‐type phenotype.

These findings have important implications for antifungal stewardship and public health policy. Routine susceptibility testing and molecular diagnostics could help prevent the inappropriate use of terbinafine, reduce treatment failures and slow the spread of resistance. Surveillance systems that monitor regional resistance trends, coupled with clinician education on interpreting MIC and mutation data, will be vital in containing this emerging threat.

## Author Contributions

T.J., Z.P.H., C.M., C.K., C.V., D.S., G.R. and J.S.K. have all had substantial contributions to conception and design, acquisition of data, or analysis and interpretation of data; drafting the article or revising it critically for important intellectual content; final approval of the version to be published; and agreement to be accountable for all aspects of the work in ensuring that questions related to the accuracy or integrity of any part of the work are appropriately investigated and resolved.

## Disclosure

Dr. Gemma Robertson is an employee of Melbourne Pathology. Prof. Johannes Kern is an Editorial Board member of Australasian Journal of Dermatology and a co‐author of this article. To minimise bias, they were excluded from all editorial decision‐making related to the acceptance of this article for publication.

## Conflicts of Interest

The authors declare no conflicts of interest.

## Supporting information


**Appendix S1:** Database: Ovid MEDLINE(R) ALL <1946 to January 20, 2023>.

**Appendix S2:** Included Studies and Data Extraction Sheet.

## Data Availability

The data that support the findings of this study are available from the corresponding author upon reasonable request.
