# Supplementary material for: Emerging Terbinafine Resistant Trichophyton Dermatophytosis, Testing Options and Alternative Treatments: A Systematic Review
Source: Australas J Dermatol. 2025 Jul 24;66(7):377–87. doi: 10.1111/ajd.14575 (PMC12633701; doi:10.1111/ajd.14575)
Supplement: Supplementary file 1 — Appendix S1: Database: Ovid MEDLINE(R) ALL <1946 to January 20, 2023>. [file AJD-66-377-s001.docx]

Database: Ovid MEDLINE(R) ALL <1946 to January 20, 2023>

Search Strategy:

--------------------------------------------------------------------------------

1 exp Tinea/ (12073)

2 (dermatophyt* or epidermophyt* or ringworm or tinea or trichophyt* or trychophyt* or

endodermophyt*).tw,kf. (16817)

3 (T mentagrophytes or T interdigitale or T rubrum or T indotineae).tw,kf. (1916)

4 Trichophyton/ (5648)

5 or/1-4 (21196)

6 Terbinafine/ (2036)

7 terbinafine.tw,kf. (2990)

8 (alm 12834 or alm12834 or binasil or curasil or daskil or dermafin or exifine or htu 520 or

htu520 or interb).tw,kf. (2)

9 (labijin or lamicosil or lamifen or lamisil or lamisila or lamisilate or lamisilatt or lamisildermgel

or lamisilmono or lamisilonce or lapiderm or lespo or micoset or micosil or "mob 015" or

mob015).tw,kf. (114)

10 (namuzol or nri 1002 or nri1002 or p 3058 or p3058 or penecure or sf 86 327 or sf 86327 or

sf86 327 or sf86327 or sulmedin).tw,kf. (24)

11 ("tdt 067" or tdt067 or terbiderm pr terbifin or terbinafin or terbinex or terbisil or terekol or

terfine or termisil or tersilat).tw,kf. (35)

12 or/6-11 (3377)

13 exp Drug Resistance, Fungal/ (7113)

14 (resistan* or refractory or recalcitran* or susceptibil* or toleran* or intransigen*).tw,kf.

(1839372)

15 ((hard or difficult*) adj3 (treat* or cure)).tw,kf. (31727)

16 (TRF-resistan* or terbinafine-resistan*).tw,kf. (97)

17 or/13-16 (1864986)

18 5 and 12 and 17 (467)

19 exp Microbial Sensitivity Tests/ (150161)

20 (amplified refractory mutation system-polymerase chain reaction or ARMS PCR).tw,kf.

(1292)

21 (random amplified polymorphic DNA or RAPD).tw,kf. (8325)

22 ((analys* or sequenc*) adj3 (Internal Transcribed Spacer or ITS)).tw,kf. (55399)

23 (antifungal susceptibility test* or AST or AFST).tw,kf. (31672)

24 Squalene Monooxygenase/ (318)

25 (squalene epoxidase or squalene monooxygenase).tw,kf. (579)

26 or/20-25 (96888)

27 5 and 12 and 26 (198)

28 18 or 27 (512)

***************************

Database: Embase <1974 to 2023 January 20>

Search Strategy:

--------------------------------------------------------------------------------

1 exp Tinea/ (9962)

2 (dermatophyt* or epidermophyt* or ringworm or tinea or trichophyt* or trychophyt* or

endodermophyt*).tw,kf. (18129)

3 (T mentagrophytes or T interdigitale or T rubrum or T indotineae).tw,kf. (2740)

4 Trichophyton/ (2961)

5 or/1-4 (21977)

6 Terbinafine/ (8934)

7 terbinafine.tw,kf. (4405)

8 (alm 12834 or alm12834 or binasil or curasil or daskil or dermafin or exifine or htu 520 or

htu520 or interb).tw,kf. (15)

9 (labijin or lamicosil or lamifen or lamisil or lamisila or lamisilate or lamisilatt or lamisildermgel

or lamisilmono or lamisilonce or lapiderm or lespo or micoset or micosil or "mob 015" or mob015).tw,kf. (925)

10 (namuzol or nri 1002 or nri1002 or p 3058 or p3058 or penecure or sf 86 327 or sf 86327 or

sf86 327 or sf86327 or sulmedin).tw,kf. (57)

11 ("tdt 067" or tdt067 or terbiderm pr terbifin or terbinafin or terbinex or terbisil or terekol or

terfine or termisil or tersilat).tw,kf. (84)

12 or/6-11 (9297)

13 exp Antifungal Resistance/ (6218)

14 (resistan* or refractory or recalcitran* or susceptibil* or toleran* or intransigen*).tw,kf.

(2366324)

15 ((hard or difficult*) adj3 (treat* or cure)).tw,kf. (48005)

16 (TRF-resistan* or terbinafine-resistan*).tw,kf. (131)

17 or/13-16 (2404360)

18 5 and 12 and 17 (779)

19 exp Microbial Sensitivity Test/ (13602)

20 (amplified refractory mutation system-polymerase chain reaction or ARMS PCR).tw,kf.

(2081)

21 (random amplified polymorphic DNA or RAPD).tw,kf. (9493)

22 ((analys* or sequenc*) adj3 (Internal Transcribed Spacer or ITS)).tw,kf. ( 60942)

23 (antifungal susceptibility test* or AST or AFST).tw,kf. (64680)

24 Squalene Monooxygenase/ (796)

25 (squalene epoxidase or squalene monooxygenase).tw,kf. (668)

26 or/19-24 (150762)

27 5 and 12 and 26 (297)

28 18 or 27 (855)

***************************

Database: Global Health <1910 to 2023 Week 03>

Search Strategy:

--------------------------------------------------------------------------------

1 (dermatophyt* or epidermophyt* or ringworm or tinea or trichophyt* or trychophyt* or

endodermophyt*).ti,ab. (21172)

2 (T mentagrophytes or T interdigitale or T rubrum or T indotineae).ti,ab. (6291)

3 or/1-2 (21411)

4 terbinafine.ti,ab. (2354)

5 (alm 12834 or alm12834 or binasil or curasil or daskil or dermafin or exifine or htu 520 or

htu520 or interb).ti,ab. (2)

6 (labijin or lamicosil or lamifen or lamisil or lamisila or lamisilate or lamisilatt or lamisildermgel

or lamisilmono or lamisilonce or lapiderm or lespo or micoset or micosil or "mob 015" or mob015).ti,ab. (101)

7 (namuzol or nri 1002 or nri1002 or p 3058 or p3058 or penecure or sf 86 327 or sf 86327 or

sf86 327 or sf86327 or sulmedin).ti,ab. (24)

8 ("tdt 067" or tdt067 or terbiderm pr terbifin or terbinafin or terbinex or terbisil or terekol or

terfine or termisil or tersilat).ti,ab. (18)

9 or/4-8 (2390)

10 (resistan* or refractory or recalcitran* or susceptibil* or toleran* or intransigen*).ti,ab.

(445977)

11 ((hard or difficult*) adj3 (treat* or cure)).ti,ab. (5339)

12 (TRF-resistan* or terbinafine-resistan*).ti,ab. (66)

13 or/10-12 (449158)

14 3 and 9 and 13 (319)

15 (amplified refractory mutation system-polymerase chain reaction or ARMS PCR).ti,ab. (474)

16 (random amplified polymorphic DNA or RAPD).ti,ab. (3691)

17 ((analys* or sequenc*) adj3 (Internal Transcribed Spacer or ITS)).ti,ab. (9940)

18 (antifungal susceptibility test* or AST or AFST).ti,ab. (16742)

19 (squalene epoxidase or squalene monooxygenase).ti,ab. (191)

20 or/15-19 (30799)

21 3 and 9 and 20 (146)

22 14 or 21 (352)

***************************

Database Name: CENTRAL

Search Name: Terbinafine

Date Run: 24/01/2023

ID Search Hits

#1 ((dermatophyt* or epidermophyt* or ringworm or tinea or trichophyt* or trychophyt* or endodermophyt*)):ti,ab,kw (Word variations have been searched) 1426

#2 ((T mentagrophytes or T interdigitale or T rubrum or T indotineae)):ti,ab,kw (Word variations have been searched) 46

#3 #1 or #2 1430

#4 (terbinafine):ti,ab,kw (Word variations have been searched) 591

#5 ((alm 12834 or alm12834 or binasil or curasil or daskil or dermafin or exifine or htu 520 or htu520 or interb)):ti,ab,kw (Word variations have been searched) 9

#6 ((labijin or lamicosil or lamifen or lamisil or lamisila or lamisilate or lamisilatt or lamisildermgel or lamisilmono or lamisilonce or lapiderm or lespo or micoset or micosil or "mob 015" or mob015)):ti,ab,kw (Word variations have been searched) 89

#7 ((namuzol or nri 1002 or nri1002 or p 3058 or p3058 or penecure or sf 86 327 or sf 86327 or sf86 327 or sf86327 or sulmedin)):ti,ab,kw (Word variations have been searched) 93

#8 #4 or #5 or #5 or #7 684

#9 ((resistan* or refractory or recalcitran* or susceptibil* or toleran* or intransigen*)):ti,ab,kw (Word variations have been searched) 147930

#10 (((hard or difficult*) N3 (treat* or cure))):ti,ab,kw (Word variations have been searched) 22

#11 ((TRF-resistan* or terbinafine-resistan*)):ti,ab,kw (Word variations have been searched) 0

#12 #9 or #10 or #11 147950

#13 #3 #8 #12 37

#14 ((amplified refractory mutation system-polymerase chain reaction or ARMS PCR)):ti,ab,kw (Word variations have been searched) 2775

#15 ((random amplified polymorphic DNA or RAPD)):ti,ab,kw (Word variations have been searched) 88

#16 (((analys* or sequenc*) N3 (Internal Transcribed Spacer or ITS))):ti,ab,kw (Word variations have been searched) 51

#17 ((antifungal susceptibility test* or AST or AFST)):ti,ab,kw (Word variations have been searched) 8916

#18 ((squalene epoxidase or squalene monooxygenase)):ti,ab,kw (Word variations have been searched) 4

#19 #14 or #15 or #16 or #17 or #18 11748

#20 #3 #8 #19 7

#21 #13 or #20 39

ClinicalTrials.gov

Tinea terbinafine 34

WHO ICTRP

Tinea AND terbinafine 42
